# Supplementary material for: Colorectal adenoma presence is associated with decreased menaquinone pathway functions in the gut microbiome of patients undergoing routine colonoscopy
Source: PLoS One. 2026 Mar 11;21(3):e0344050. doi: 10.1371/journal.pone.0344050 (PMC12978465; doi:10.1371/journal.pone.0344050)
Supplement: Attachment — Submitted filename: Response to Reviewers.docx [file pone.0344050.s002.docx]

**Cover letter**

**Manuscript ID:** PONE-D-25-58757
**Title:** *Colorectal adenoma presence is associated with decreased menaquinone pathway functions in the gut microbiome of patients undergoing routine colonoscopy*

Dear Jad El Masri,

We sincerely thank you for the careful evaluation of our manuscript and for the opportunity to submit a revised version. We appreciate the thoughtful and constructive comments provided by the academic editor and reviewers, which were very helpful in improving the clarity and presentation of our work. Below, we respond to each of the editorial requirements in detail.

**1. PLOS ONE style requirements**
The manuscript has been revised to comply with PLOS ONE formatting and style guidelines. File naming and manuscript structure now follow the provided PLOS ONE templates.

**2. Role of the funder**
The following statement has been included in the cover letter and manuscript: “The funders had no role in study design, data collection and analysis, decision to publish, or preparation of the manuscript.”

**3. Data Availability Statement (DOI / accession numbers)**
We thank the editor for highlighting this requirement. The Data Availability Statement has been updated to include the repository name, accession number, and a direct access link. All metagenomic sequencing data generated in this study are publicly available in the European Nucleotide Archive (ENA) under accession number **PRJEB79034** (https://www.ebi.ac.uk/ena/browser/view/PRJEB79034).

**4. Reviewer-recommended citations**
Reviewers did not recommend to add any other citation.

**5. Reference list**
The reference list has been reviewed for accuracy and completeness. No retracted articles are cited. Any updates to the references have been noted in the responses to the reviewers.

Further, we provide our response to the reviewers.

**Response to Reviewer #1**

We thank the reviewer for the positive evaluation of our study and for the thoughtful and constructive comments. We are pleased that the reviewer found the study design, statistical approach, and overall presentation appropriate, and we appreciate the helpful suggestions for improving clarity.

Regarding the sample numbers, we agree that the original wording may have caused confusion. The total number of enrolled participants was 136. After quality control, 123 samples were retained for taxonomic profiling, whereas functional pathway analysis was performed on 135 samples, as functional profiling has different quality and coverage requirements. We have revised the relevant sections of the Results to clearly distinguish between the number of enrolled participants and the number of samples included in each analysis, thereby improving clarity and consistency throughout the manuscript.

In addition, we have revised the Abstract to remove the redundant mention of statistically significant beta diversity differences, as suggested by the reviewer.

We thank the reviewer again for these valuable comments, which have helped us to improve the clarity of the manuscript.

**Response to Reviewer #2**

We thank the reviewer for the careful reading of our manuscript and for the constructive and helpful suggestions. We appreciate the opportunity to clarify and improve several aspects of the study. Our responses to each comment are provided below.

**1. Study design in the Abstract**

We thank the reviewer for this suggestion. The Abstract has been revised to explicitly state the study design as a cross-sectional case–control study, which we agree will help readers more clearly understand the study framework.

**2. Redundancy in reporting beta diversity results**

We thank the reviewer for pointing out this redundancy. The Abstract has been revised to remove the repeated statement regarding statistically significant beta diversity differences, improving clarity and conciseness.

**3. Clarification of inconclusive findings in previous studies (Introduction, lines 86–90)**

We thank the reviewer for this helpful comment. We have revised the Introduction to clarify in what way previous findings are inconclusive. Specifically, we now note that published studies differ in the reported direction and magnitude of microbial diversity changes, as well as in the identification of taxa associated with adenoma presence. In addition, methodological heterogeneity - such as differences in sequencing platforms, analytical pipelines, lesion characteristics, and population backgrounds - has contributed to inconsistent and sometimes conflicting results.

**4. Dietary and medication data (Methods section)**

We thank the reviewer for raising this important point. To minimize the impact of major dietary- and medication-related confounders on gut microbiome composition, patients who had used antibiotics or probiotic supplements within one month prior to inclusion were excluded from the study. In addition, none of the included participants reported adherence to a vegan or vegetarian diet at the time of enrolment. Although detailed dietary intake data were not systematically collected, these criteria were applied to reduce extreme dietary patterns and medication exposures known to influence gut microbial composition substantially. The Methods section has been revised accordingly to clarify these points.

**5. Consistency between Abstract and Results regarding significant taxa**

We thank the reviewer for highlighting this issue. The Abstract has been revised to clarify that the reported genus-level differences (e.g., *Bacteroides* and *Prevotella*) represent descriptive trends observed prior to multiple-testing correction. We now clearly distinguish these descriptive patterns from the species-level analysis, in which only *UBA7597* sp003448195 remained statistically significant after multivariable adjustment and FDR correction. This revision ensures full consistency between the Abstract and the Results section.

We thank the reviewer again for the thoughtful and constructive feedback, which has helped us to improve the clarity of the manuscript.

**Response to Reviewer #3**

We thank the reviewer for the positive assessment of our manuscript and for recognizing the importance of the research question and the value of the real-world data presented. We also appreciate the constructive suggestions, which have helped us to improve the clarity and precision of the manuscript. Our detailed responses are provided below.

**1. Contradiction regarding beta diversity**

We thank the reviewer for identifying this inconsistency. The statement in the Discussion section has been corrected. While alpha diversity did not differ significantly between adenoma and control groups, beta diversity analysis demonstrated statistically significant differences in overall microbial community structure, as reported in the Results section (p = 0.0002). The revised Discussion text now accurately reflects these findings and is fully consistent with the Results.

**2. Clarification of the term “metabolic issues”**

We thank the reviewer for this helpful suggestion. The term “metabolic issues” was indeed too vague and has been revised. We now specify that changes in gut microbiota may contribute to colorectal cancer through altered microbial metabolic functions, in addition to chronic inflammation and immune dysfunction. This revised wording more precisely reflects the mechanisms discussed and aligns with the functional results of our study.

**3. Insufficient figure legends**

We thank the reviewer for highlighting this issue. All figure legends have been revised and expanded to provide clear, self-contained descriptions. The updated legends now specify the analysis performed, sample groups and sizes, statistical approaches, and key observations for each figure, thereby improving interpretability and readability.

**4. Broad conclusion in the Abstract**

We thank the reviewer for this comment. The Conclusions section of the Abstract has been revised to provide a more specific and data-driven summary of the main findings, highlighting the observed functional differences in microbial metabolic pathways associated with colorectal adenomas.

**5. Weak and overly general background in the Abstract**

We appreciate this suggestion. The Background section of the Abstract has been strengthened to better emphasize the clinical relevance of colorectal adenomas as early neoplastic lesions and to clearly outline the existing knowledge gap regarding microbiome functional alterations at the adenoma stage, which this study aims to address.

We thank the reviewer again for the insightful ad constructive feedback, which has significantly improved the clarity, consistency, and overall quality of the manuscript.

We believe that the revised manuscript adequately addresses all editorial and reviewer comments and has been significantly improved as a result. We appreciate your time and consideration and look forward to your further evaluation.

Kind regards,
Ilona Vilkoite, on behalf of all authors
02.02.2026.
